# Supplementary material for: Experimental Insights into the Interplay between Histone Modifiers and p53 in Regulating Gene Expression
Source: Int J Mol Sci. 2023 Jul 3;24(13):11032. doi: 10.3390/ijms241311032 (PMC10342072; doi:10.3390/ijms241311032)
Supplement: Supplementary file 1 [file ijms-24-11032-s001.zip › ijms-2443440-supplementary.pdf]

Table S1. Multiple drugs within the DNMT, HDAC, EZH2 and IDH1/2 inhibitor categories have been approved by regulatory authorities, including the US FDA and other government bodies, for the treatment of diverse cancer types**.**

	Name	Target categories	References	Links
**1**	5-Azacytidine	DNMT	[102]	https://www.accessdata.fda.gov/drugsatfda_docs/label/2022/050974s034lbl.pdf
**2**	Decitabine (5-aza-2′-deoxycytidine)	DNMT	[103]	https://www.accessdata.fda.gov/drugsatfda_docs/label/2018/021790s021lbl.pdf
**3**	Vorinostat (Zolinza)	HDAC	[104,105]	https://rsc.niaid.nih.gov/sites/default/files/Vorinostat%20%28Zolinza%29%20PI_dated%20July%202022.pdf
**4**	Romidepsin (Istodax)	HDAC	[97]	https://www.accessdata.fda.gov/drugsatfda_docs/label/2021/022393Orig1s016lbl.pdf
**5**	belinostat (Beleodaq)	HDAC	[106]	https://www.accessdata.fda.gov/drugsatfda_docs/label/2014/206256lbl.pdf
**6**	Panobinostat (Farydak)	HDAC	[107]	https://www.accessdata.fda.gov/drugsatfda_docs/label/2015/205353s000lbl.pdf
**7**	Tazemetostat (Tazverik)	EZH2	[108]	https://www.accessdata.fda.gov/drugsatfda_docs/label/2020/213400s000lbl.pdf
**8**	Enasidenib (Idhifa)	isocitrate dehydrogenase-2 inhibitor (indirectly modulate DNA methylation)	[109]	https://www.accessdata.fda.gov/drugsatfda_docs/label/2017/209606s000lbl.pdf
**9**	Ivosidenib (Tibsovo)	isocitrate dehydrogenase-1 inhibitor (indirectly modulate DNA methylation)	[110]	https://www.accessdata.fda.gov/drugsatfda_docs/label/2021/211192_s008lbl.pdf

References

97. Schnell, A.P., et al., HDAC inhibitors Panobinostat and Romidepsin enhance tax transcription in HTLV-1-infected cell lines and freshly isolated patients' T-cells. Front Immunol, 2022. 13: p. 978800.

102. Christman, J.K., 5-Azacytidine and 5-aza-2'-deoxycytidine as inhibitors of DNA methylation: mechanistic studies and their implications for cancer therapy. Oncogene, 2002. 21(35): p. 5483-95.

103. Buocikova, V., et al., Decitabine-induced DNA methylation-mediated transcriptomic reprogramming in human breast cancer cell lines; the impact of DCK overexpression. Front Pharmacol, 2022. 13: p. 991751.

104. Shankar, S., et al., Suberoylanilide hydroxamic acid (Zolinza/vorinostat) sensitizes TRAIL-resistant breast cancer cells orthotopically implanted in BALB/c nude mice. Mol Cancer Ther, 2009. 8(6): p. 1596-605.

105. Siegel, D., et al., Vorinostat in solid and hematologic malignancies. J Hematol Oncol, 2009. 2: p. 31.

106. Tuncer, Z., et al., Apoptotic effect of Belinostat (PXD101) on MCF-7 cancer cells. Annals of Medical Research, 2021. 28(5).

107. Meng, W., et al., Enhanced efficacy of histone deacetylase inhibitor panobinostat combined with dual PI3K/mTOR inhibitor BEZ235 against glioblastoma. Nagoya J Med Sci, 2019. 81(1): p. 93-102.

108. Bekric, D., et al., Evaluation of Tazemetostat as a Therapeutically Relevant Substance in Biliary Tract Cancer. Cancers (Basel), 2023. 15(5).

109. Khalil, A.F., et al., Discovery of novel enasidenib analogues targeting inhibition of mutant isocitrate dehydrogenase 2 as antileukaemic agents. J Enzyme Inhib Med Chem, 2023. 38(1): p. 2157411.

110. Reinbold, R., et al., Resistance to the isocitrate dehydrogenase 1 mutant inhibitor ivosidenib can be overcome by alternative dimer-interface binding inhibitors. Nat Commun, 2022. 13(1): p. 4785.
